# Obesity and Cardiovascular Disease: The Emerging Role of Inflammation

**DOI:** 10.3389/fcvm.2021.768119

**Published:** 2021-10-25

**Authors:** Rana Khafagy, Satya Dash

**Affiliations:** ^1^Department of Pharmacy, The Hospital for Sick Children, Toronto, ON, Canada; ^2^Banting & Best Diabetes Centre, University of Toronto, Toronto, ON, Canada; ^3^Department of Medicine, University Health Network, Toronto, ON, Canada

**Keywords:** obesity, cardiovacsular disease(s), inflammation, athersclerosis, genetic pathway

## Abstract

Obesity is a growing public health challenge across the globe. It is associated with increased morbidity and mortality. Cardiovascular disease (CVD) is the leading cause of mortality for people with obesity. Current strategies to reduce CVD are largely focused on addressing traditional risk factors such as dyslipidemia, type 2 diabetes (T2D) and hypertension. Although this approach is proven to reduce CVD, substantial residual risk remains for people with obesity. This necessitates a better understanding of the etiology of CVD in people with obesity and alternate therapeutic approaches. Reducing inflammation may be one such strategy. A wealth of animal and human data indicates that obesity is associated with adipose tissue and systemic inflammation. Inflammation is a known contributor to CVD in humans and can be successfully targeted to reduce CVD. Here we will review the etiology and pathogenesis of inflammation in obesity associated metabolic disease as well as CVD. We will review to what extent these associations are causal based on human genetic studies and pharmacological studies. The available data suggests that anti-inflammatory treatments can be used to reduce CVD, but off-target effects such as increased infection have precluded its broad therapeutic application to date. The role of anti-inflammatory therapies in improving glycaemia and metabolic parameters is less established. A number of clinical trials are currently ongoing which are evaluating anti-inflammatory agents to lower CVD. These studies will further clarify whether anti-inflammatory agents can safely reduce CVD.

## Introduction

Obesity is a chronic disease which increases mortality and morbidity and has reached epidemic proportions (1, 2). Recent data estimates that roughly 604 million adults and 108 million children worldwide are obese (3). This has led to an increase in obesity-related comorbidities including cardiovascular disease, type 2 diabetes (T2D), fatty liver disease, dementia, osteoarthritis, obstructive sleep apnea, and several cancers (3–5). Cardiovascular disease (CVD) is of particular concern due to its significant mortality, strain on healthcare systems, and loss of labor productivity (6). Despite therapeutic progress, CVD is the leading cause of mortality in people with obesity, accounting for ~70% of deaths in people with obesity (5, 6).

The increased risk of CVD, and in particular atherosclerotic CVD (ACVD), in people with obesity is to a large extent mediated by traditional established risk factors such as insulin resistance, dyslipidemia, T2D, hypertension, and obstructive sleep apnea (OSA) (7). Despite improved treatments to target these traditional risk factors, people with obesity remain at risk of ACVD, suggesting that additional factors play a role (7). Recent data indicates that inflammation is an important contributor to ACVD (8, 9).

Notably, obesity is associated with chronic low-grade inflammation, which is a plausible mediator of the increased CVD seen in people with obesity (10–14). Here we will review the association between inflammation, obesity and ACVD. As genetically validated therapeutic targets have increased likelihood of success, we will specifically focus on the genetic evidence for a causal association between inflammation and cardio-metabolic disease.

## The Association Between Obesity and Established CVD Risk Factors

Obesity is a chronic disease in which excess adiposity impairs health (15). It is associated with insulin resistance, dyslipidemia, T2D, hypertension, and OSA, which are established CVD risk factors (7, 14). Although conventionally defined by a body mass index (BMI; weight in kilograms divided by square of height in meters) >30, this does not uniformly stratify patients at risk of cardiometabolic disease (16, 17). In contrast, waist-to-hip ratio (WHR) is a better predictor of both metabolic disease and myocardial infarction compared to BMI (16, 17). In a recent observational study from Holland which included participants from multiple ethnic groups (African Surinamese, South Asian Surinamese, Turkish, Moroccan, Ghanaian, and Dutch Caucasian), WHR was the most reliable predictor of T2D, overall and across ethnic groups, in both men and women (18). The receiver operated curves (ROC) for WHR was 0.78 in men and 0.81 for women (18). The ROC for BMI was 0.68 and 0.74 in men and women, respectively (18). Observational data also indicates that the odds ratio for myocardial infarction significantly increased for every successive WHR quintile (1.15, 1.39, 1.9, and 2.52, respectively) (17). Risk of myocardial infarction for those in the top two quintiles of BMI was 7.7%, compared to 24.3% for the top two quintiles of WHR (17). For each 1 standard deviation increase in WHR, the odds ratio of myocardial infarction increased by 1.37, even following adjustment for BMI (17). In contrast, the odds ratio increased by 1.10 for BMI and 1.02 when adjusted for WHR (17).

## Obesity and Insulin Resistance in T2D

**Figure 1:**
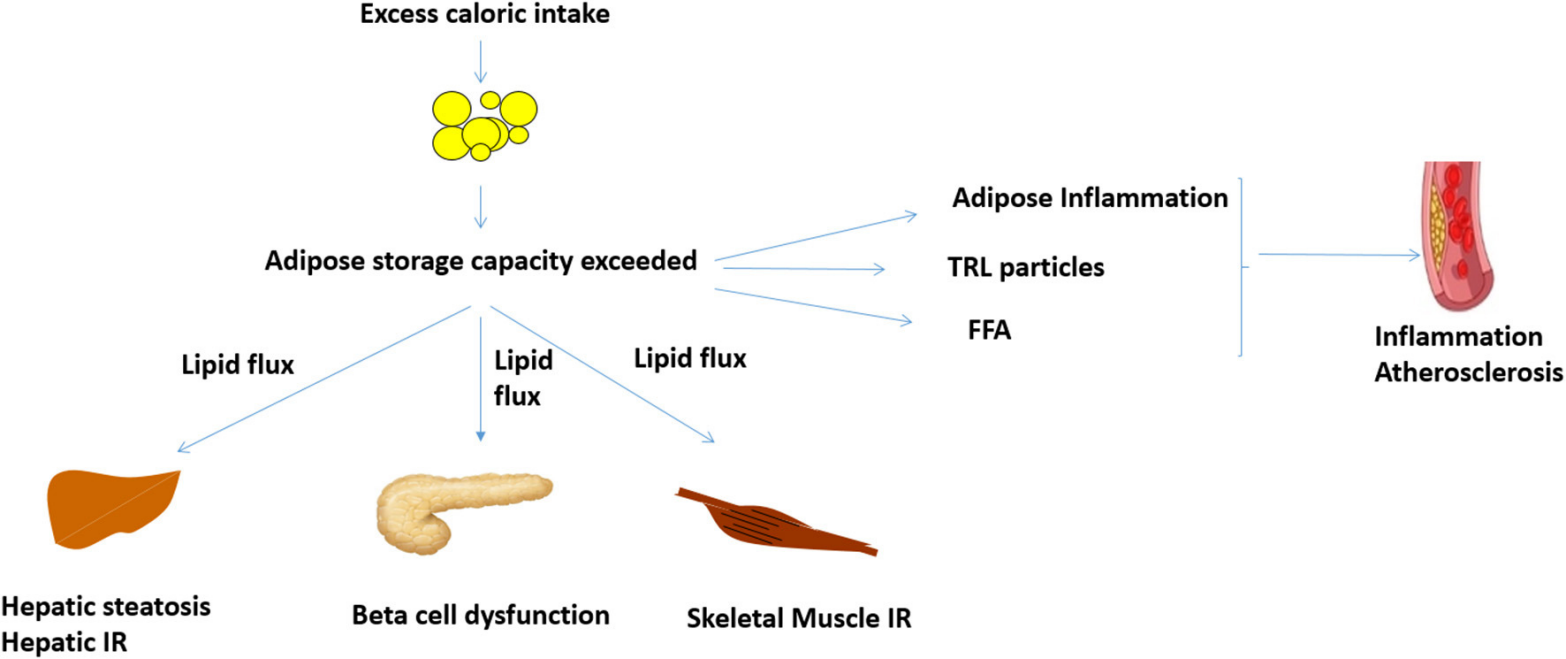
Proposed mechanisms linking obesity, inflammation and cardiovascular disease. When adipose storage capacity is exceeded, increased lipid flux, and ectopic lipid in liver, skeletal muscle, and pancreas reduce insulin sensitivity and beta cell function. Compromised adipose storage is also associated with adipose and systemic inflammation, which can potentially potentiate atheroma development. Increased TRL and FFA, which are features of insulin resistance, may also increase inflammation and atheroma development. *FFA, free fatty acids; IR, insulin resistance; TRL, triglyceride rich lipoproteins*.

Increased WHR, a predictor of insulin resistance and T2D, is associated with increased centripetal adiposity and/or lack of femoro-gluteal adiposity (16, 19). Genetic analyses suggest that these are causal associations mediated by reduced adipose storage capacity (7, 20). Weight gain in the presence of reduced adipose storage capacity leads to ectopic lipid deposition in the liver, skeletal muscle, and pancreas and increase in visceral adipose tissue (Figure 1) (13, 14). The BMI threshold at which this occurs is variable and influenced by age, ethnicity, sex, and genetic factors (13, 14). Although obesity rates are higher in women, pre-menopausal women are protected from metabolic disease (21–26). Conversely, men develop metabolic disease at lower BMI (21–26).

Ectopic lipid in liver and skeletal muscle has been causally implicated in insulin resistance via lipid intermediaries such as diacylglycerol and ceramides (27, 28). Pancreatic lipid deposition likely impairs beta cell function (29). Weight loss of 5–10% can reduce ectopic lipid, thus improving insulin sensitivity and glycemica (30). Greater weight loss of ~15% or more, either through reduced caloric intake or bariatric surgery (the most efficacious weight loss treatment), can reverse ectopic lipid deposition and potentially reverse insulin resistance and T2D (29, 31–33).

## Obesity and Insulin Resistance in Dyslipidemia

Patients with obesity and insulin resistance frequently have elevated triglyceride (TG), triglyceride rich lipoproteins (TRL), low high density lipoproteins (HDL), and increased small dense low density lipoproteins (LDL) (34). The increase in TG and TRL is likely mediated by compensatory hyperinsulinaemia, secondary to insulin resistance in the presence of increased lipid flux to liver and intetsine (34). Consequently, hepatic lipogenesis, and production of TRL from liver (very low-density lipoprotein secreted in the fasted and post-prandial state) and intestine (chylomicrons secreted after meals) increases (34). Increased TG/TRL results in triglyceride enriched HDL which has enhanced hepatic clearance which reduces reverse cholesterol transport and lowers HDL. Increased TRL also yields smaller dense LDL particles (13). TRL undergo lipolysis to yield remnant particles. Both TRL remnants and small dense LDL likely have atherogenic properties (13). Human genetic studies have consistently implicated TRL as a causative risk factor for CVD (35–39).

Weight loss improves dyslipidemia in patients with insulin resistance (13). Weight loss through lifestyle intervention, pharmacotherapy, and bariatric surgery have been shown to lower plasma triglycerides (TG) and increase plasma HDL but whether this translates to lower CVD is not conclusively established (40–42) Pharmacotherapy to reduce plasma TG, which does not always result in reduced TRL/TRL remnant particle number, has not consistently translated to reduced CVD (13).

## Obesity and Insulin Resistance in Hypertension

Obesity is estimated to contribute to ~70% of the risk for primary/essential hypertension (14, 16). Mechanical effects of visceral fat on natriuresis, leptin-mediated sympathetic nervous system activation as well as increased renin-aldosterone action likely contribute to obesity-associated hypertension (43–45).

Obesity is associated with a 2-fold increased risk in OSA and prevalence of OSA in those with obesity has been reported to be ~45% (46, 47). Treatment of OSA with continuous positive airway pressure (CPAP) therapy induces small but significant improvement in hypertension (48). Weight loss through lifestyle changes, the medication liraglutide and bariatric surgery attenuates many of the underlying pathological processes contributing to hypertension and improves/resolves hypertension, especially early in the course of the disease before end organ damage (40, 41, 43, 49).

## Obesity, Insulin Resistance and Inflammation

As alluded to earlier, compromised adipose storage capacity in the setting of weight gain is causally associated with cardiometabolic disease. WHR is a better predictor of compromised adipose storage and cardiometabolic disease than BMI (16, 19). Compromised adipose storage is associated with adipose hyperplasia and hypertrophy and hypoxia with apoptosis (50, 51). This is associated with recruitment of inflammatory cells including macrophages, neutrophils, and lymphocytes (50). Compromised adipose storage is also associated with increased visceral adipose tissue and visceral adipose inflammation (50). Adipose tissue macrophages in insulin resistant states are polarized to a more inflammatory phenotype and secrete inflammatory cytokines including tumor necrosis factor-alpha (TNF-alpha), interleukin-1beta and interleukin-6 (Table 1) (50, 52). Administration of TNF-alpha in mice induces insulin resistance, while attenuation of TNF-alpha with genetic or pharmacologic manipulation protects against metabolic dysfunction (52). TNF-alpha has been shown to increase the activity of kinases such as c-Jun N-terminal kinases (JNK 1 and 2) and I kappa B Kinase (IKK), which phosphorylate insulin receptor substrate at serine residues to impair insulin action (52). Adipose inflammation is also associated with recruitment of neutrophils which release neutrophil elastase, promoting further increase in adipose tissue macrophage infiltration (52). Innate lymphoid cells, CD4+ helper T cells, cytotoxic CD8+ cells and innate-like T cells further propagate inflammation with secretion of inflammatory cytokines including, TNF-alpha and gamma-interferon (50). In animal models, gamma-interferon has been implicated in impaired insulin signaling, reduced adipogenesis, and adipose storage via JAK-STAT signaling (50). Depleting these sub-populations of lymphocytes in obesity in mouse models is associated with protection against metabolic disease (50).

**Table 1:** Critical regulators of inflammation in obesity and CVD (50, 52).

	**Impact on metabolic function**	**Impact on atherosclerosis**
Free fatty acid	-Contributes ectopic lipid deposition and insulin resistance and type 2 diabetes	-Activates NLRP3 inflammasome and TLR4 in macrophages
IL-6	-Impairs insulin sensitivity and increases T2D risk in genetic analyses	-Secreted by macrophages to further increase inflammation in atheromas
PAI-1	-FFA increases production -Elevated levels found in individuals with abdominal fat accumulation	-Increases risk of intravascular thrombus and CVD by inhibiting tPA and contributing to fibrinolysis and atherothrombosis
TNF-alpha	-Secreted by macrophages in adipose tissue. Implicated in reduced insulin signaling in animal models	-Secreted by macrophages and inflammatory cells in atheromas to further increase inflammation -Causally implicated in CVD in Mendelian randomization analysis

*FFA, free fatty acid; IL, interleukin; IR, insulin resistance; PAI-1, plasminogen activator inhibitor-1; T2D, type 2 diabetes; TG, triglycerides; TNF, tumor necrosis factor; tPA, tissue plasminogen activator; WC, waist circumference*.

Free fatty acids, which are increased in obesity and insulin resistance, can directly impact inflammation. Palmitate, which is increased in high fat fed mice, activates the NACHT, LRR, and PYD domain-containing protein 3 (NLRP3) inflammasome protein complex which secretes caspase-1 (52). This results in cleavage and secretion of active IL-1beta and Il-18 from macrophages (52, 53). In addition to insulin resistance, IL-1 beta has been implicated in impaired insulin secretion and T2D (53). Fatty acid can also promote inflammation via activating TLR4 (toll like receptor 4), which in turn can activate macrophages and TNF-alpha production (52).

## Impact of Inflammation on Atheroma Development

Inflammation plays an important role in the development of an atheroma (8, 9). Blood vessels have three layers: tunica intima (facing the lumen), tunica media, and tunica adventitia. Vessel wall damage in the tunica intima layer and endothelial dysfunction are early steps in the pathogenesis of atherosclerosis (8). Under these circumstances, endothelial express adhesion molecules such as vascular cell adhesion molecule-1 (VCAM-1) and chemoattractant proteins such as of monocyte chemoattractant protein 1 (MCP-1), which recruit inflammatory cells including monocytes and lymphocytes to the endothelium and can further propagate inflammation by secretion of cytokines such as interleukin-1, interleukin-6, TNF-alpha, and colony stimulating factor 1 (8). Monocytes recruited to vessel mature into macrophages and take up cholesterol particles to form foam cells (8). The cytokine milieu promotes vascular smooth cell proliferation within the intima, which with the secretion of extracellular matrix gives further propagates atheroma development (8). Animal data indicates that vascular smooth muscle cells from the tunica media can migrate to the intima and undergo metaplasia and acquire foam cell markers (8, 9). Apoptosis and ineffective clearance of phagocytes and other inflammatory entities promotes the development of a necrotic core in the atherosclerotic lesion (8, 9). Superficial erosion of the plaque due to loss of the endothelial monolayer can result in entrapped neutrophils releasing “neutrophil extracellular traps,” which further propagate inflammation and thrombus formation with healing leading to stenosis of the vessel (8, 9). Plaque rupture activates a coagulation cascade and thrombus formation with acute ischemia/infarction (8).

Obesity and associated inflammatory processes can potentially modulate these steps in atheroma development. Vessel wall damage and recruitment of inflammatory cells is likely enhanced under conditions of systemic inflammation (8). TRL, which are increased with insulin resistance, promote inflammation directly given their apolipoprotein CIII content and by delivering cholesterol to macrophages in the atheroma (8). In *in vitro* studies, TRL remnant particles upregulate the expression MCP-1, a key step in the recruitment of monocytes to vascular endothelial cells (54). It also upregulates a number of cellular adhesion molecules such as VCAM-1 (vascular cell adhesion molecule-1) and ICAM-1 (intracellular adhesion molecule-1). These processes facilitate retention of monocytes and formation of foam cells (54). TRL particles promote vascular smooth muscle cell proliferation *in vitro*, a key step in plaque progression. In animal models, insulin resistance, and associated hyperinsulinemia is associated with selective insulin resistance in the vasculature; insulin signaling via Phosphoinositide 3-kinase (PI3K) is impaired but insulin signaling via mitogen-activated protein kinase (MAPK) signaling is increased (13). The increased MAPK signaling promotes vasoconstriction due to endothelin-1 secretion, proliferation of vascular smooth muscle cells, secretion of pro-coagulant factors such as Plasminogen activator inhibitor-1 (PAI-1) and secretion of chemo-attractant proteins and cell adhesion molecules which promote recruitment of macrophages (13).

To what extent obesity associated inflammation modulates atheroma development in people with established CVD is not established. Circulating C-reactive protein, a marker of inflammation, is a predictor of CVD and is higher in people with obesity, in particular centripetal adiposity (55). Among individuals with high CRP free of CVD, those with obesity have higher CRP and higher coronary artery calcium scores and carotid artery intima thickness (55). However, this association appeared to be independent of CRP (55). Data on other inflammatory markers were unavailable—notably Mendelian randomization indicates that raised CRP *per se* does not cause CVD (56).

## Clonal Hematopoiesis of Indeterminate Potential

Somatic mutations in hematopoietic stem cells leads to clonal expansion of hematopoietic cells and has been implicated in various hematological malignancies (54, 57, 58). The majority of patients with clonal hematopoiesis do not develop malignancy (clonal hematopoiesis of indeterminate potential) (54, 57, 58). This is however associated with increased risk of CVD in part due to increased secretion of pro-inflammatory cytokines, including IL-6, with greater recruitment and retention of macrophages in plaques and increased vascular smooth muscle proliferation contributing to ACVD and heart failure (54, 57, 58).

## Genetic Evidence for a Potential Role for Obesity Associated Inflammation in ACVD

Circulating cytokines: As alluded to above, obesity and insulin resistance are associated with increased circulating concentration of inflammatory cytokines including TNF-alpha, IL-1beta, IL-6, and Il-18 (54, 57, 58). Mendelian randomization studies assess the genetic association between a trait and a downstream outcome. Such associations are suggestive of a causal link, providing the genetic instrument does not affect an intermediary trait that can influence the downstream outcome (59). Genetically-determined increase in TNF have been associated with ACVD, suggesting a causal link (60). However, it also protects against malignancy (60). Whether this impacts insulin resistance or T2D is not established. Genetically-determined increase in IL-6 action is associated with both increased risk for T2D and CVD, suggesting shared underlying etiology (61, 62). Data from genetic and pharmacologic studies of IL-1 receptor modulation have not been consistent. Genetically-determined IL-1 receptor antagonist was surprisingly has been associated with increased CVD; whether this is due to dual IL-1 alpha and beta reduction is not clear (62). Genetically modulated IL-1 receptor activity does not impact T2D risk (63). Notably, pharmacological IL-1beta blockade has been shown to reduce CVD in a large randomized control trial with no effect on progression of glycemia (64). Genetically-determined IL-18 is not associated with either T2D or CVD (62, 63).

CHIP: In the Women's Health Initiative study, CHIP increased with increased BMI in post-menopausal women (highest in those with BMI>30 vs. BMI 27–30 kg/m^2^ compared to normal weight women) (65). This suggests obesity may be associated with increased CHIP. CHIP may also be increased in patients with T2D (66). A potential contributor to CHIP in T2D and obesity may be the adipokine leptin (67). Circulating leptin concentration is proportional to fat mass and increase/decrease with fat gain/loss (68). Leptin increases haematopoiesis and activates Janus kinase 2 (JAK2), a critical node for CHIP (66, 69). In mice, reduction in leptin via exercise-induced weight loss reduced CHIP (67).

In summary, the available evidence suggests increased circulating IL-6 and TNF-alpha, which are features of obesity associated insulin resistance, likely causally increase risk of CVD. CHIP, a more recently reported CVD risk factor, may be increased in T2D and obesity.

## Pharmacological Evidence Supporting a Role of Inflammation in T2D and CVD

Recently, there has been pharmacologic evidence for the association between inflammation and CVD (Table 2). The JUPITER trial concluded that individuals with increased levels of the inflammatory biomarker C-reactive protein (CRP) responded to rosuvastatin pharmacotherapy and had significant decreases in major cardiovascular events, regardless of presence of dyslipidemia (71). Statins are known to lower cholesterol, as well as high-sensitivity CRP (71). Healthy adults with a high CRP treated with rosuvastatin were found to have, on average, a 47% lower risk of myocardial infarction, stroke, or death from cardiovascular causes compared to those who did not receive statin therapy (71). This confirms that CVD is an inflammatory disorder and that inflammatory markers can be utilized to stratify patients, independent of traditional risk factors such as LDL. ~40% of patients in the trial had evidence of metabolic syndrome; to what extent centripetal adiposity/obesity modulated atherosclerosis and the response to statin treatment is not known (71).

**Table 2:** Pharmacologic therapies for CVD targeting inflammatory pathway.

**Drug**	**Trial (Author)**	**Mechanism**	**Study findings**	**Comments**
Anakinra	VCU-ART3 Abbate et al. (70)	Decrease IL-1 receptor	CRP AUC decreased with treatment in patients with STEMI (median 67 vs. 214; *p* <0.001)	Significantly decreased death, new onset HF or death/hospitalization for HF as well; effets short-term (rebound CRP and IL-6 upon stopping); not supported by genetic studies
Canakinumab	CANTOS Ridker et al. (64)	Decreasing IL-1b	Nonfatal MI, stroke or CV death decreased with the 150mg dose (HR 0.83; *p =* 0.005)	Independent of dyslipidemia; patients had high CRP at baseline; higher incidence of fatal infection compared to placebo; no significant impact on all-cause mortality
Colchicine	CALCOT Tardif et al. (71)	Decrease CRP, NLRP3 inflammasome inhibitor	CV death, resuscitated cardiac arrest, MI, stroke, or urgent hsopitalization for angina requiring coronary revascularization decreased with treatment (HR 0.77; *p =* 0.02)	Significant GI side effects
Darapladib	SOLID-TIMI 52 O'Donoghue et al. (72)	Decrease lp-PLA2	No significant difference in major coronary events with treatment (HR 0.99; *p =* 0.78)	Genetic studies inconsistent; lp-PLA2 did not decrease inflammatory markers
Low dose IL-2	LILACS Zhao et al. (73)	Promotes regulatory T-cells	Results pending	Effective in preclinical data; more selective T-cell regulators than Aldesleukin being developed
Methotrexate	CIRT Ridker et al. (74)	Dihydrofolate reducatase inhibitor	Nonfatal MI, stroke or CV death not significantly changed with treatment (HR 0.96; *p =* 0.91)	Treatment did not decrease inflammatory markers; pathway may not be relevant
Rosuvastatin	JUPITER Ridker et al. (71)	Decrease high-sensitivity CRP	MI, stroke or death from CV causes decreased with treatment (HR 0.56; p <0.00001)	Independent of dyslipidemia
Tocilizumab	ASSAIL-MI Broch et al. (75)	Anti-IL-6 receptor antibody	Myocardial salvage in acute STEMI larger with treatment (difference 5.6; *p =* 0.04)	No significant difference in infarct size between treatment and placebo; non-specific blocker of IL-6 signalling
Varespladib	VISTA-16 Nicholls et al. (76)	Decrease sPLA2	CV death, nonfatal MI, nonfatal stroke and unstable angina did not significantly decrease with treatment (HR 1.25; *p =* 0.08)	Trial stopped early for greater risk of MI with treatment; non-specific treatment; pathway not supported by Mendelian randomization
Xilonix	El Sayed et al. (77)	Anti-IL-1a antibody	MACE did not significantly change with treatment (9% vs. 24%; *p =* 0.22)	Limited clinical data available; did not lower CRP

*AUC, area under the curve; CRP, C-reactive protein; CV, cardiovascular; GI, gastrointestinal; HF, heart failure; HR, hazard ratio; IL, interleukin; Lp-PLA2, lipoprotein-associated phospholipase A2; MACE, major adverse cardiovascular events; MI, myocardial infarction; S-PLA2, secretory phospholipase A2; STEMI, ST-segment elevation myocardial infarction*.

A number of anti-inflammatory agents have been evaluated in CVD outcomes trials. These agents are not known to affect weight or metabolic disease and thus any effects are independent of weight loss and metabolic status (64, 74, 78–81). The CANTOS trial evaluated the role of IL-1 beta antagonism on the incidence of T2D and CVD. CVD decreased by ~15% in patients with elevated hsCRP, an effect seen with and without T2D (64, 78). These finding are scientifically important as they represent the first convincing evidence that a strategy that targets a specific inflammatory pathway reduces CVD. The long term feasibility of this therapy remains to be determined given potential side effects, including sepsis, and cost (64). Intriguingly, although canakinumab reduced CVD in patients with T2D, it did not affect glycaemia in the long term (78). It did not prevent incident T2D in normoglycaemic patients and those with pre-T2D. Furthermore, the magnitude of reduction in CVD risk in patients with T2D compared to those without (78). These results suggest that IL-1b contributes to CVD risk in patients with inflammation but likely does not play a major role in the etiology of T2D.

The CALCOT trial evaluated the use of colchicine in individuals with a recent myocardial infarction (79). It concluded that low-dose colchicine resulted in a significantly lower risk of cardiovascular events compared to placebo post-myocardial infarction (79). However, there was an increase in incidence of pneumonia in the treatment group (79). These CVD benefits of colchicine were confirmed in the Low Dose Colchicine 2 (LoDoCo2) study, although there was a trend toward increased non-CVD death (82). Colchicine inhibits tubulin polymerization and microtubule generation and its role in CVD is linked to inhibition of the NLRP3 inflammasome (79). Although initial data suggested colchicine may be beneficial for improving insulin sensitivity and glycemia, this has not been subsequently confirmed (79–81).

In the CIRT study, low-dose methotrexate, an anti-inflammatory agent, did not reduce levels of interleukin-1b, interleukin-6 or CRP, nor did it result in a difference in cardiovascular events compared to placebo (74). Methotrexate inhibits dihydrofolate reductase which may not be of relevance in CVD (9, 74). Darapladib, an lp-PLA2 (Lipoprotein-associated phospholipase A2) inhibitor, and varespladib, an sPLA2 (secretory phospholipase A2) inhibitor, have not been promising either in clinical trials (9). They target phospholipase A2 which are secreted by inflammatory cells and postulated to contribute to atherosclerosis. Notably genetic studies of this pathway in humans have not consistently showed an association with CVD (9).

There is considerable interest in utilizing tocilizumab, an IL-6 receptor antagonist, for treating CVD and T2D as this is a genetically validated target as discussed above (9). However, a potential concern with IL-6 inhibition is an increase in LDL due to reduced clearance (83).

In summary, there is growing evidence that reducing inflammation can lower incident CVD but off-target effects, in particular infection/sepsis, are a concern. In contrast, we do not have convincing evidence yet that anti-inflammatory therapies lead to sustained reduction in T2D and metabolic disease (72, 73, 75–77, 84).

## Impact of Weight Loss

Weight loss can potentially reduce inflammation and improve multiple CVD risk factors. In the LOOK-AHEAD trial, modest weight loss associated with intensive lifestyle changes was associated with improvement in cardiometabolic parameters in patients with T2D but overall no CVD benefit was seen (42). *Post-hoc* analyses suggest that those who achieved sustained weight loss of 10% or more had a reduction in CVD (85). Similarly, weight loss through pharmacotherapy improves cardiometabolic parameters, but whether this translates to benefits in major CVD outcomes is unknown (40, 86). GLP-1 receptor agonists (GLP-1RA) have beneficial cardiovascular outcomes in patients with T2D (86). The exact mechanisms have not been delineated but are likely independent of glucose lowering as not all glucose lowering drugs prevent CVD (87). Further, the effects are likely independent of blood pressure lowering and weight loss as the GLP-1RA albiglutide reduces CVD despite no significant reduction in weight or blood pressure (88). Animal models suggest that GLP-1 may have beneficial effects on vascular inflammation (89). Higher doses of GLP-1 analogs are now being used and evaluated as weight loss agents (40, 90). Whether these agents will improve cardiovascular outcomes remains to be seen. The SELECT study will evaluate the effect of 2.4 mg once weekly of semaglutide on heart disease and stroke in patients with obesity and CVD (NCT03574597).

To date, bariatric surgery remains the most efficacious weight loss treatment and improves multiple metabolic parameters, including adipose tissue and systemic inflammation (51, 70, 91, 92). Retrospective data analysis suggests that bariatric surgery is associated with reduced major adverse cardiovascular outcomes in patients with obesity both with and without T2D (70, 91–93). The extent to which this is mediated by reduced inflammation secondary to weight loss will require more detailed mechanistic studies.

## Effect of Weight Loss on Inflammatory Markers

As discussed earlier, increased WHR is likely causally associated with increased adipose and systemic inflammation. Consistent with that weight loss is associated with reduced adipose and systemic inflammation. Bariatric surgery is associated with decreases in CRP and interleukin-6 concentrations in proportion to weight less, however, TNF-alpha levels did not change (51, 94) Despite this, insulin resistance was not normalized and some adipose pathology remained post-surgery (51). Lifestyle weight loss interventions, with or without statins, have also been found to decrease CRP but whether this translates to reduced CVD is not established (95). Liraglutide treatment, which is known to reduce CVD in people with T2D, has been associated with reductions in inflammatory markers, but to what extent this is mediated by weight loss is unknown (40).

## Conclusion

Although considerable progress has been made in reducing the burden of CVD, it remains the leading cause of mortality in people with obesity. Thus, further therapies are needed to reduce the burden of CVD. Inflammation is a key mediator of atherosclerosis and can potentially be targeted for reduction in CVD; its role in treating T2D and metabolic disease is less established. However, to date, lack of efficacy, and off-target effects have limited the broad utility of anti-inflammatory treatments. The emergence of more “omics” data will likely identify further anti-inflammatory targets. Whether this translates to reduced cardiometabolic disease remains to be seen. In the interim, we await more data from current clinical trials evaluating anti-inflammatory agents to reduce CVD. There is emerging observational data that substantial weight loss, through bariatric surgery, may reduce CVD. The extent to which this is mediated by reduction in inflammation remains to be determined.

## Author Contributions

RK and SD wrote the manuscript. All authors contributed to the article and approved the submitted version.

## Funding

SD was funded by the Canadian Institute for Health Research, Diabetes Canada, Heart & Stroke Foundation of Canada and Banting & Best Diabetes Center (DH Gales Family Charitable Foundation New Investigator Award and Reuben & Helene Dennis Scholar in Diabetes).

## Conflict of Interest

The authors declare that the research was conducted in the absence of any commercial or financial relationships that could be construed as a potential conflict of interest. SD has received speaker fees/consultant fees from Novonordisk and Eli Lilly.

## Publisher's Note

All claims expressed in this article are solely those of the authors and do not necessarily represent those of their affiliated organizations, or those of the publisher, the editors and the reviewers. Any product that may be evaluated in this article, or claim that may be made by its manufacturer, is not guaranteed or endorsed by the publisher.
